# Eclectic Department

**Published:** 1855-06

**Authors:** 


### ECLECTIC DEPARTMENT,

### AND SPIRIT OF THE MEDICAL PERIODICAL PRESS.

---

Prof. John T. Metcalfe publishes, in the N. Y. Med. Times, some remarks on the treatment of pneumonia, from which we extract the conclusion. Of 12 cases described, 2 proved fatal.—Ed. Journal.

Of the two fatal cases, one was fairly moribund when first seen. It occured in the person of a confirmed inebriate, and attacked the upper part of the lung, as is not unfrequently seen in the pneumonia of sufferers from delirium tremens. Treatment seemed to produce no good effect; nor do I think venesection or calomel would have proved useful in the case (VI)

The other fatal result (Case II.) was in the person of a man with completely developed spanaemia, the result of a malarious fever of the Isthmus, in whom the pulmonary inflammation was complicated with universal bronchitis, and whose extremely prostrated state allowed nothing more heroic than calomel to be used. This seemed productive of no good, and he ultimately died of the secondary bronchitis.

In the other cases, the treatment, with the exception of that which had reference to particular symptoms, was negative, so far as it was especially addressed to the *cure* of the disease. Diet, repose, change of posture, and relief by vomiting free accumulated bronchial mucus, constituted the whole.

It often occurs that patients enter the New York and Bellevue Hospitals, whose histories are such as have been related in the foregoing cases. They have been taken ill with a chill, pain in the side, cough and fever, which have disabled them from work. Looking upon their illness as a "bad cold," from which a few days' rest, with a dose of oil or salts, will relieve them, they take to bed, and remain, in expectation of recovery, from four or five days to a week, when, finding no improvement in their condition, they have recourse to the Hospital. The account given of themselves, taken with the results of physical exploration, leaves no doubt that inflammation of the lungs is the disease in question.

On referring to those who have written systematically on the pathology and therapeutics of this malady, and who, with students and young practitioners, constitute authority, I have been struck with the almost uniform tendency to consider pneumonia as a disease which threatens life in a most serious manner, and which requires, for its successful management, the energetic employment of antiphlogistic remedies. True, there are, by some, exceptions made in these cases of very old people—of those who have been attacked whilst greatly debilitated, and in epidemics of typhoid pneumonia; but there is still, no doubt, a very general recognition of the necessity for opposing what is looked upon as a formidable disease by heroic remedies.

By some authors, implicit reliance is placed upon early resort to the lancet, to scarified cups, and to the production of the constitutional effect of mercury on the system, by inducing ptyalism. By others, the Rasorian administration of tartar emetic is regarded as the means most likely to insure a certain and speedy cure of the disease. It would not, perhaps, be erroneous to state that one or other of these, or a combination of the two, constitute the basis of English and American therapeutics.

Observation, by comparing the progress of cases in which bleeding and mercurialization had been trusted to, with those in which rest, proper regulation of ventilation, and appropriate diet, together with such *juvantia* as might be indicated in any particular case, has seemed to convince me that in pneumonia, as we see it in New York, entirely too much stress has been laid on the necessity of having recourse to the former therapeutical course. Nor can I resist the conviction that, in other localities, a careful study of the natural history of the disease would tend to lower the general estimate in which blood-letting and calomel are held, as potent agents in *curing* pneumonia.

Skoda, drawing not a drop of blood, employing solely *extractum graminis*, or a few grains of nitre, and in a few instances corrosive sublimate, lost three, only, of forty-four patients, whose average age was between twenty-five and twenty-six years.

Varrentrapp, following the example of Wacherer and Baumgärtner, teaches us how the disease may be successfully brought to convalescence by giving no medicine internally except chloroform vapor, applied by inhalation to the mucous membrane of the lungs. His results show a mortality of one in twenty of those treated in this way, or 5 per cent., agreeing very closely with those of Wacherer, Baumgärtner, Helbing, and Schmidt, who, in one hundred and ninety-three cases lost nine, or 4.25 per cent., on the chloroform treatment.

In 1848, Dr. Dietl, of Cracow, published the result of one hundred and eighty-nine cases of pneumonia, treated by diet and rest alone. Of these he lost only 7.4 per cent. In 1853, he published a second pamphlet, detailing the success he met with in seven hundred and fifty or sixty cases, treated entirely by hygienic and dietetic means. I regret that this monograph has been mislaid. I can only state that the general result accords with that above mentioned, and served to confirm in his mind the superiority of the expectant, over the methods by venesection, calomel, or tartar emetic.

Skoda, Varrentrapp, and Dietl, had all previously commenced their practice by treating inflammation of the lungs according to the traditional means last mentioned. None of them, although good diagnosticians and men of great intelligence, have been otherwise than satisfied with the result of their change of practice. Skoda is not more active in reality than Dietl, in his treatment; nor can it be truly said that the inhalation of chloroform does any thing in the way of curing the disease. They all trust to the well-known potency of nature, when allowed to act without restraint or interference; and if they had never made any other contribution to medical science, these physicians would deserve the gratitude of the profession for the information, so little possessed before their researches, and so inadequately diffused at present, that pneumonia has a natural tendency to get well when let alone, in a great majority of cases in which it attacks healthy subjects; and that more harm than good results, as a general rule, from the employment of what are called heroic remedies in its therapeutics.

Had Dietl, Skoda, or Varrentrapp, experimented with the infinitesimal puerilities of Hahneman, instead of adopting the course actually pursued, we might easily imagine the result on their minds, if they had been as susceptibly constituted as those of many men who, having compared the results of Sangradaism with those of Hahnemanism have joyfully embraced the latter creed, without ever entertaining a suspicion that truth was to be found neither in one extreme nor the other, but where it usually exists, in the happy medium.

The above is the substance of some clinical remarks delivered at the New York Hospital, when reviewing the treatment of pneumonia, during the months of January, February, and March, of the present year. I have not touched on the questions as to whether there may not be means employed which will relieve particular symptoms, such as pain, cough, and dyspnœa, from excessive secretion and from intestinal accumulations. I am very sure that we do possess such *juvantia*, and that the result of common experience has left no doubt of their value. What I have endeavored to inculcate is the fact that, in the disease under consideration, there is no *absolute necessity* for resorting to extreme measures.

In examining candidates for posts in the House Staff of Bellevue Hospital, since the year 1847, I have had graduates from nearly all sections of the United States among the applicants, and have been impressed with the fact, that nearly all of them have been imbued with the firm conviction that inflammation of the lungs was a most dangerous affection, one which, if not promptly attacked with the lancet and calomel, or by antimonial medicines, would be very apt to prove fatal to the patient. To such, it has given me satisfaction to show the progress of the disease in cases which have entered the service with all the signs and symptoms of pneumonia, which have had no medicine previously to admission, and for the management of which, attention to diet, ventilation, posture, and a few teaspoonfuls of mucilage of gum arabic in the day, have constituted the sole treatment. Nearly all have been persons of the laboring class, whose return to health was a matter of importance. How different their condition, after the subsidence of the disease, from that of others who have been copiously bled, or who had undergone "a course of mercury."

I do not speak of the power of venesection, as an ectrotic, in the first stage of the disease. My experience has reference to it as seen in hospitals, and as it is almost always met with in private practice. It would be instructive to have a number of such cases detailed, with the evidence that the disease really was the one in question.—*N. Y. Med. Times.*

